# Non-invasive prediction of NAFLD severity: a comprehensive, independent validation of previously postulated serum microRNA biomarkers

**DOI:** 10.1038/s41598-018-28854-4

**Published:** 2018-07-13

**Authors:** Mireia López-Riera, Isabel Conde, Guillermo Quintas, Laia Pedrola, Ángela Zaragoza, Judith Perez-Rojas, Mario Salcedo, Salvador Benlloch, José V. Castell, Ramiro Jover

**Affiliations:** 1 Hepatología Experimental, IIS Hospital La Fe, Valencia, Spain; 2 Medicina Digestiva, Sección Hepatología, Hospital La Fe, Valencia, Spain; 3 Health and Biomedicine, Leitat Technological Center, Barcelona, Spain; 4 Unidad de Genómica, Servicio de Secuenciación, IIS Hospital La Fe, Valencia, Spain; 5 Anatomía Patológica, Sección Hepatología, Hospital La Fe, Valencia, Spain; 6 Medicina Interna, Hospital La Fe, Valencia, Spain; 7 Centro de Investigación Biomédica en Red de Enfermedades Hepáticas y Digestivas (CIBERehd), Instituto de Salud Carlos III, Madrid, Spain; 8 Departamento de Bioquímica y Biología Molecular, Facultad de Medicina, Universidad de Valencia, Valencia, Spain

## Abstract

Liver biopsy is currently the only reliable method to establish nonalcoholic fatty liver disease (NAFLD) severity. However, this technique is invasive and occasionally associated with severe complications. Thus, non-invasive diagnostic markers for NAFLD are needed. Former studies have postulated 18 different serum microRNA biomarkers with altered levels in NAFLD patients. In the present study, we have re-examined the predictive value of these serum microRNAs and found that 9 of them (miR-34a, -192, -27b, -122, -22, -21, -197, -30c and -16) associated to NAFLD severity in our independent cohort. Moreover, miR-192, -27b, -22, -197 and -30c appeared specific for NAFLD, when compared with patients with drug-induced liver injury. Preliminary serum RNAseq analysis allowed identifying novel potential miRNA biomarkers for nonalcoholic steatohepatitis (NASH). The classification performance of validated miRNAs (and their ratios) for NASH was better than that reached by AST, whereas for advanced fibrosis prediction miRNAs did not perform better than the FIB-4 algorithm. Cross-validated models combining both clinical and miRNA variables showed enhanced predictivity. In conclusion, the circulating microRNAs validated demonstrate a better diagnostic potential than conventional serum markers to identify NASH patients and could complement and improve current fibrosis prediction algorithms. The research in this field is still open.

## Introduction

Nonalcoholic fatty liver disease (NAFLD) is one of the most important causes of liver disease worldwide and will likely emerge as the leading reason of end-stage liver disease in the near future, thus placing a growing strain on health-care systems. NAFLD has a global prevalence of 24% and involves a high risk of liver-related morbidity and mortality along with metabolic comorbidities^1^. NAFLD covers a wide spectrum of histologic lesions, ranging from isolated hepatic steatosis (nonalcoholic fatty liver; NAFL) to nonalcoholic steatohepatitis (NASH), the latter characterized by the presence of lobular inflammation and hepatocyte ballooning, with or without fibrosis^2^. NASH, with a prevalence among biopsied NAFLD patients of 59%^3^, and advanced fibrosis have been associated with a risk of evolution to cirrhosis and hepatocellular carcinoma, and increased liver-related and cardiovascular mortality^2,4^. Therefore, identifying patients with NASH is a key clinical issue.

The diagnosis of NAFLD severity (i.e. NASH and advanced fibrosis) is established from a liver biopsy, where hepatosteatosis, lobular inflammation, ballooning and fibrosis are semi-quantitatively scored. Thus, liver biopsy allows for grading and staging, and provides information about prognosis. Two semiquantitative histological scoring systems are currently used. One is the NAFLD activity score (NAS) proposed by the NASH Clinical Research Network^5,6^, that grades steatosis, hepatocellular ballooning, and lobular inflammation. Summation of these grades of active injury in NAS scores is recommended in clinical trials. More recently the Steatosis, Activity, Fibrosis (SAF) algorithm has been proposed by Bedossa *et al*.^7,8^ in compliance with the American Association for the Study of Liver Disease. This comprises the same histological characteristics, but with steatosis not included in the activity score (ballooning + lobular inflammation). Fibrosis stage is also scored separately in both systems.

The histopathological analysis of liver biopsy is central in the diagnosis of NASH^7^. However, liver biopsy is an invasive procedure and has several limitations: sampling error is common as NAFLD do not affect the liver uniformly, histology interpretation is subjective, and biopsies are costly and associated with complications (e.g. pain, serious bleeding, injury to other organs and, in rare cases, death)^9^. For these reasons, several clinical prediction rules and blood-based biomarkers have been developed as attractive and affordable non-invasive alternatives for identification of patients at risk for NASH and advanced fibrosis. These range from standard clinical and biochemical parameters to markers that reflect specific molecular mechanisms underlying the pathogenesis and progression of NAFLD, including inflammation, oxidative stress and apoptosis^10^.

Recently, the discovery of a fundamental role of microRNAs (miRNAs) in disease pathogenesis and their stable presence in biological fluids has led to extensive investigations on the potential role of miRNAs as emerging non-invasive biomarkers for disease diagnosis and prognosis^11^. miRNAs are small (∼22 nucleotide-long) non-protein coding RNA species involved in post-transcriptional regulation of gene expression. In serum/plasma, miRNAs are stable because they are protected from degradation by extra-cellular vesicles (such as exosomes), miRNA binding proteins (such as argonaute) and high-density lipoproteins^11^. The increasing enthusiasm in the use of circulating miRNAs in clinical practice is explained by their ability to reflect the physiological/pathological state of the tissue they are derived from. Thus, circulating miRNAs may be regarded as fingerprints of the affected tissue. Moreover, they are powerful tools to understand disease biology^12^.

The trend on miRNA biomarkers has not gone unnoticed to researchers focusing on NAFLD diagnosis and 14 studies on circulating miRNA biomarkers in NAFLD patients have been published in the last years^12–26^. Surprisingly, the miRNAs proposed in these independent studies show a very limited overlap, and only the liver specific miR-122 is consistently postulated as a potential circulating NAFLD biomarker. Such a lack of agreement is remarkable and may come from limitations such as too few miRNAs examined, different technical approaches, small study populations, no biopsy-proven severity and no validation; or it may also be that this promise remains a work in progress. Only few studies have attempted de novo miRNA discovery by using omics approaches^12,16,18^, whereas most others have focused on particular miRNAs based on previous knowledge, such as miRNA relevance on disease-related pathways or miRNA level alterations in animal models or human liver samples.

In the present study we have reexamined the predictive value for NASH and fibrosis of all the serum microRNAs postulated so far, and compared their performance with that of conventional serum-based clinical biomarkers and predictive algorithms, in a cohort of patients with biopsy proven NAFLD. Analysis of results clarifies which of the postulated miRNAs validate as robust predictive biomarker for the identification of NAFLD patients at risk.

## Results

### A search for miRNAs previously identified as circulating biomarkers in NAFLD patients

A systematic literature search for studies involving serum/plasma miRNAs in NAFLD patients, aimed at identifying miRNAs that predict/associate/correlate with disease and/or severity (i.e. NASH and fibrosis) returned 14 published studies (Table 1). We did not select studies exploring miRNA signatures in just human liver nor those focusing only on animal models. We did also not include studies analysing circulating miRNAs that associate only with specific clinical features within NAFLD patients, as coronary artery disease or obesity (see for example^20^).

**Table 1:** Summary of clinical studies on serum miRNAs as predictive biomarkers in NAFLD.

PatientsTraining + (Validation)	Biopsy proven	Approach	Up-regulated miRNAs	Down-regulated miRNAs	Correlation (with)	AUROC (predict)	Reference Year
19 Controls 34 NAFLD [16 NAS ≥ 5]	Yes	RT-PCR	-122, -34a (NASH) -16 (NAFLD)		-122, -34a (ALT, AST, NAS & Fib.)	-122/-16 /ALT → 0.93/0.96/0.91 (NAFL) -34a/-122→ 0.75/0.70 (NASH)	^22^ 2011
311 Controls 92 NAFLD	No	RT-PCR	-21, -34a, -122, -451 (NAFLD)		-122 (Steatosis)		^17^ 2013
90 + (80) Controls 152 + (103) NAFLD	Yes	Illumina sequencing/RT-PCR	-122, -1290, -27b, -192 (NAFLD)			4-miR-panel/ALT → 0.89 /0.79 (NAFLD)	^16^ 2014
20 Controls 20 NAFLD	Yes	RT-PCR		-181d, -99a, -197, -146b (NAFLD)	-197 (Inflamm.)	-197/-146b/-181d/-99a → 0.77/0.75/0.86/0.76 (NAFLD)	^21^ 2014
52 NAFLD	Yes	RT-PCR miR-122	-122 (steatosis)	-122 (Fib.)	-122 (Fib.)	-122/hyaluronate/ collagen IV → 0.82/0.74/0.72 (Fib.)	^26^ 2014
16 + (19) Controls 16 + (30) NAFL 16 + (47) NASH	Yes	miRNAs PCR-based array (n = 84)/RT-PCR	-122, -192, -375 (NASH) -122 (Fib.)		-122, -192 (AST, GGT, TG & CK18)	-122/-192/-375 → 0.69/0.68/0.72 (NAS > 5) -122/ALT/AST/CK18 → 0.71/0.66/0.68/0.65 (NASH) 0.61/0.60/0.64/0.50 (Fib.)	^12^ 2015
61 Controls 50 NAFL 87 NASH	Yes	RT-PCR	-122, -192, -21 (NASH)		-122, -192 (ALT & CK18) -21 (Inflamm.)	3-miR panel/CK18/ALT → 0.81/0.81/0.77 (NASH)	^20^ 2015
12 Controls 25 NAFLD	No	RT-PCR miR-21		-21 (NAFLD)			^15^ 2015
37 Controls 17 NAFL 31 NASH	Yes	RT-PCR	-122, -192, -34a (NASH) -16, -21, -146 (NAFLD)		-122, -192, -34a (Inflamm. & Ballooning) -16 (Fib.)	-34a/ALT/CK18/FIB4 /APRI → 0.81/0.68/0.71/0.68 /0.73 (NASH) -16/FIB4/APRI → 0.71/0.84/0.85 (Fib.)	^23^ 2016
62 Controls 18 NAFLD	No	OpenArray RT-PCR System (n = 818)	-122, -34a* (NAFLD)	-331, -30c (NAFLD)			^18^ 2016
28 Controls 36 NAFLD	Yes	RT-PCR	-122, -34a (NAFLD)		-34a (VLDL & TG)	-34a/-122/ALT → 0.78/0.86/0.83 (NAFLD)	^14^ 2016
305 NAFLD (139 NAS ≥ 5)	Yes	RT-PCR miR-122	-122 (Steatosis, Inflamm., Ballooning & Fib.)		-122 (AST, ALT, Inflamm., Ballooning & Fib.)		^27^ 2016
31 Controls 27 NAFL 34 NASH	Yes	RT-PCR	-122 (NASH)		-122 (Glucose, HDL, AST, ALT, Inflamm. & Ballooning)	-122 → 0.82 (NAFLD)/0.76 (Inflamm. & Ballooning)	^19^ 2016
10 Controls 43 NAFLD	Yes	RT-PCR	-22, -29a (NASH) -663a (NAFLD)		-22, -29a (Fib.) -22 (NAS)		^24,32^ 2016/2017

Only 3 studies performed de novo discovery of miRNAs (PCR-based arrays or next-generation sequencing)^12,16,18^, whereas in all the others, the investigated miRNAs were selected based on relevance in disease-associated pathways or from previous findings in liver tissue or NAFLD models. Moreover, three of these studies focused in only one pre-selected miRNA^13,15,26,27^.

Analysis of the 14 selected studies revealed a total of 18 miRNAs that were significantly altered in NAFLD patients, or in NASH and severe fibrosis groups (Table 1). Five of these miRNAs were present in more than one study: -122 (n = 11), -34a (n = 5), -192 (n = 4), -21 (n = 3) and -16 (n = 2), whereas 13 miRNAs were described in only 1 study.

### Characteristics of the study population

The majority of the cohort (72%) was within the overweight-obese range (BMI = 25–35) with a 60% of women and with an age average of 53 years. The NL (normal liver) and NAFL groups were fairly similar, as only age and plasma glucose showed statistically significant differences (Table 2). However, the NL and NASH groups showed more dissimilarities, including differences in glucose, triglycerides (TG), ALT (alanine aminotransferase), AST (aspartate aminotransferase) and ferritin (Table 2).

**Table 2:** Baseline characteristics of the NAFLD study cohort.

	NL (n = 17)	NAFL (n = 25)	NASH (n = 50)	
Age (years)	42.9 ± 9.3	51.6 ± 10.4	57.4 ± 10.4	a,b
Sex				
Male Female	7 (41.2%) 10 (58.8%)	11 (44%) 14 (56%)	19 (38%) 31 (62%)	
Body mass index (kg/m^2^)	26.2 ± 3.6	29.6 ± 5.2	32.4 ± 4.6	b
Waist perimeter (cm)	nd	99.8 ± 17.0	107.5 ± 11.1	c
Glucose (mg/dL)	89.9 ± 10.7	118.1 ± 39.7	126.7 ± 62.3	a,b
Triglycerides (mg/dL)	106.7 ± 36.3	126.3 ± 66.3	190.8 ± 104.4	b,c
Total cholesterol (mg/dL)	203.7 ± 37.4	202.6 ± 43.6	199 ± 39.2	
HDL-cholesterol (mg/dL)	55.0 ± 18.2	52.5 ± 11.2	45.6 ± 13.4	
LDL-cholesterol (mg/dL)	128.1 ± 34.7	123.7 ± 41.5	122.9 ± 33.7	
Total bilirubin (mg/dL)	0.6 ± 0.2	0.7 ± 0.3	0.6 ± 0.3	
Albumin (g/dL)	4.5 ± 0.5	4.6 ± 0.3	4.6 ± 0.3	
Platelets (10^3/µL)	245.2 ± 75.1	260.1 ± 74.2	218.4 ± 66.5	c
ALT (IU/L)	29.8 ± 16.7	51.8 ± 32.1	64.7 ± 52.6	b
AST (IU/L)	26.0 ± 8.8	36 ± 15.1	54.6 ± 35.4	b,c
ɣ-GT (IU/L)	50.8 ± 51.4	134 ± 144.8	122.8 ± 128.1	
ALP (IU/L)	86.1 ± 32.7	106.4** ± **48.2	95.4** ± **35.5	
Protrombin (sec)	14.6 ± 2.1	14.1 ± 1.4	14 ± 1.6	
Hb (g/dL)	13.9 ± 0.9	14.7 ± 1.2	14.4 ± 1.1	
Transferrin saturation (%)	27.2 ± 9.6	26.8 ± 11.7	28.1 ± 11.0	
Ferritin (µg/L)	66.8 ± 54.7	113.1 ± 58.7	283.8 ± 325.7	b,c
HbA1C (%)	nd	6.4 ± 1.4	6.5 ± 1.3	
Insulin (µU/mL)	nd	17.4 ± 11.1	32.5 ± 37.3	c
HOMA	nd	4.7 ± 3.6	11.2 ± 16.9	c
*HISTOLOGY*				
Steatosis (%)				***
Grade 0				
Grade 1		20 (80%)	4 (8%)	
Grade 2		3 (12%)	23 (46%)	
Grade 3		2 (8%)	23 (46%)	
Ballooning (%)				***
None (0)		18 (72%)		
Moderate (1)		7 (28%)	20 (40%)	
Severe (2)			30 (60%)	
Lobular inflammation (%)				***
None (0)		13 (52%)	1 (2%)	
Moderate (1)		12 (48%)	35 (70%)	
Severe (2)			14 (28%)	
Fibrosis (%)				***
Stage 0		21 (84%)	4 (8%)	
Stage 1		4 (16%)	9 (18%)	
Stage 2			8 (16%)	
Stage 3			16 (32%)	
Stage 4			13 (26%)	
*SCORES*				
NAS (%)				***
NAS_0–2		19 (76%)		
NAS_3–4		6 (24%)	12 (24%)	
NAS_5–8			38 (76%)	
SAF activity A_0–1 A_2–3–4		25 (100%)	50 (100%)	***
NAFLD fibrosis score				***
<−1.455	15 (88.2%)	15 (60%)	18 (36%)	
−1.45/−0.675		6 (24%)	4 (8%)	
−0.675/0.676	2 (11.8%)	4 (16%)	19 (38%)	
>0.676			9 (18%)	
FIB-4				***
<1.30	14 (82.4%)	17 (68%)	16 (32%)	
>1.30	3 (17.6%)	8 (32%)	34 (68%)	
APRI				**
<1	17 (100%)	24 (96%)	35 (70%)	
>1		1 (4%)	15 (30%)	
BARD				
<2	4 (23.5%)	8 (32%)	10 (20%)	
≥2	13 (76.5%)	16 (64%)	40 (80%)	

Data are shown as mean ± standard deviation or as number of cases and %. NL, normal liver; NAFL, nonalcoholic fatty liver; NASH, nonalcoholic steatohepatitis; HDL, high-density lipoprotein; ALT, alanine aminotransferase; AST, aspartate aminotransferase; ɣ-GT, gamma-glutamyltransferase; ALP, alkaline phosphatase; Hb, Hemoglobin; HbA1C, glycosylated hemoglobin; HOMA, homeostatic model assessment; APRI, AST to platelet ratio index; FIB-4, fibrosis-4; nd, not determined. **a** NL vs NAFL, **b** NL vs NASH, **c** NAFL vs NASH, *p* < *0.05* (ANOVA or t-test); **p < *0.01*; ***p < *0.001* (Chi-square).

Regarding histology of NAFL patients, 24% presented pure steatosis, 28% presented steatosis with moderate ballooning and 48% steatosis with moderate lobular inflammation. Only 4 NAFL patient had mild fibrosis (stage 1). As expected, the 50 patients with NASH showed more severe histological features (Table 2).

### Serum level of previously postulated miRNAs and their relationship with liver expression

**Figure 1:**
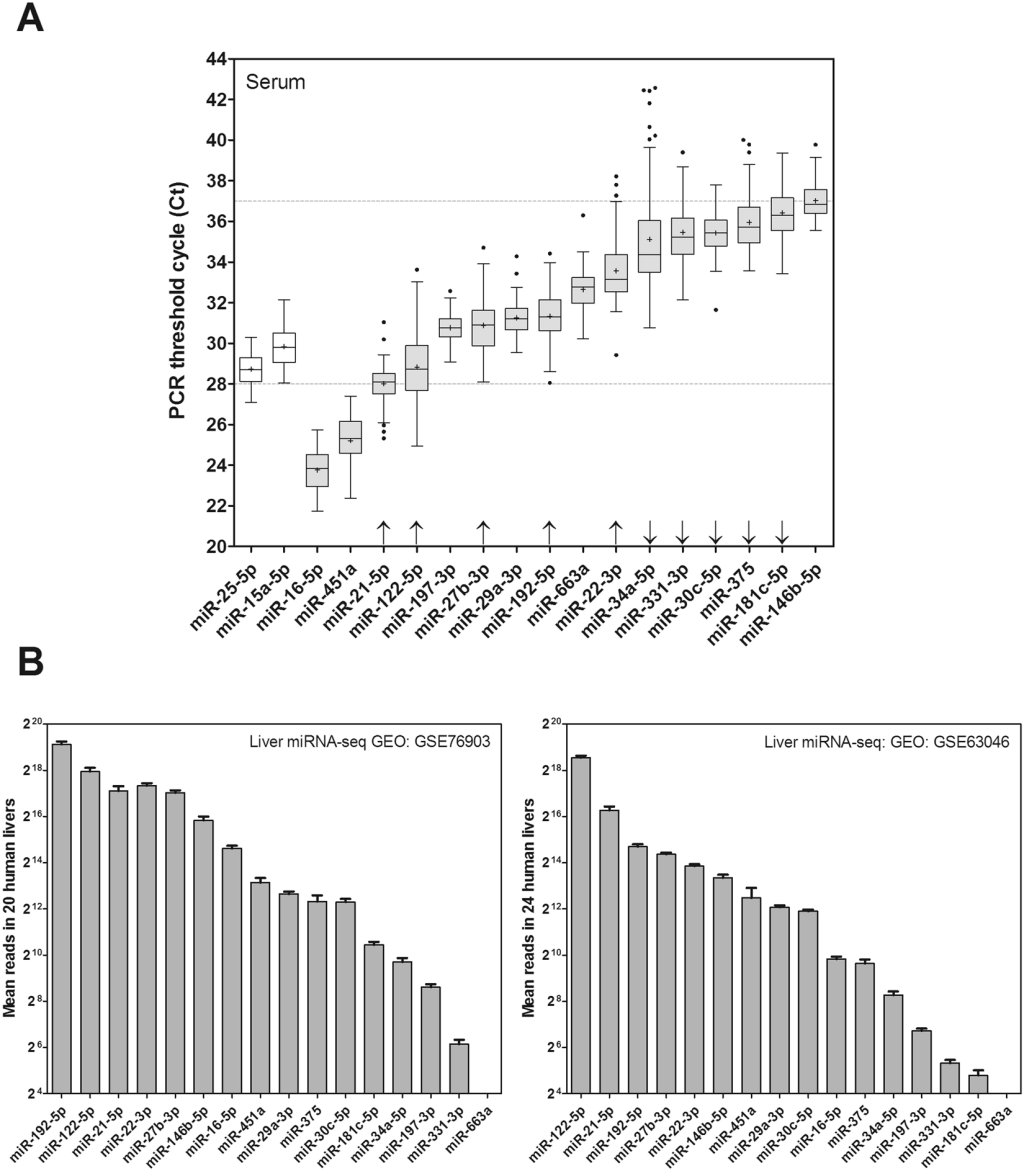
Expression of NAFLD-associated miRNAs in human serum and liver. (**A**) Total RNA was purified from 92 serum samples, and 18 specific miRNAs were amplified by RT-PCR. Results are represented as PCR threshold cycles (Ct) in box plots with whiskers extending to the nearest data points within 1.5 Inter Quartile Range (IQR). Data beyond the end of the whiskers are plotted individually (Boxplot Tukey). Horizontal dotted lines at cycles 28 and 37 delimit serum expression levels for most analysed miRNAs. Arrows above X-axis indicate miRNAs with high (up) and low (down) liver expression. (**B**) The Gene Expression Omnibus (GEO) repository was searched for datasets with non-coding RNA profiling of human livers by high throughput sequencing. Two datasets, GSE76903 29 and GSE63046 30 (Illumina HiSeq), included 20 and 24 control livers (HCC adjacent normal tissue), respectively. The normalized sequencing reads of the NAFLD-associated miRNAs were extracted, processed as mean ± SEM and ordered from higher to lower liver expression level.

We developed specific qRT-PCR (quantitative real-time polymerase chain reaction) assays for the 18 miRNAs identified in previous studies and analysed their serum levels in the entire cohort population (Fig. 1A). In our experimental conditions, two of the postulated miRNAs (-99a and -1290) failed to be amplified or were detected in only few samples with very high threshold cycles (Ct). Sixteen of the 18 miRNAs were detected with confidence (Ct <38) in most of the entire cohort (>90% of the subjects). The majority of miRNAs were detected in the range 28–37 Ct, indicating differences in the serum concentration of these miRNAs of up to 500-fold. miR-34a, the second most studied miRNA, was one of the lowest concentration in human serum, whereas miR-16 an miR-451a were the most abundant miRNAs of this set. As expected, the two miRNAs used for normalization (miR-15a and miR-25-5p) were relatively abundant in serum and showed low variability (interquartile range) (Fig. 1A).

Small RNA-sequencing allows defining the human hepatic miRNome and ranking miRNAs according to their abundance in liver tissue. We have extracted the normalized sequencing reads of the 16 miRNAs of this study in 24 + 20 samples of normal human liver tissue deposited in the Gene Expression Omnibus (GEO) Database (GSE76903^28^ & GSE63046^29^). Results reveal that, within this miRNA set, the most abundant human liver miRNAs are -122, -192, -21, -22, -27a and -146b, whereas the less abundant are -331, -197, -34a, -181c, -30c and -375 (Fig. 1B). Moreover, miR-663a was not detected in normal human livers, though it has been shown to be expressed in human hepatoma cells^24^.

Comparison of liver RNAseq data with results depicted in Fig. 1A reveals that most of the abundant liver miRNAs are detected at lower Ct in serum (arrows up), whereas most of the lower expression liver miRNAs are detected at higher Ct in serum (arrows down), which indicates a fairly good relationship between human liver and serum concentrations in this set of miRNAs. The most discrepant miRNAs were miR-16 and miR-451a (highly abundant in serum, but not in liver), which suggest important contributions from other tissues, besides liver, to the circulating level of these two miRNAs.

### Validation of serum miRNA alterations in NASH and advanced fibrosis

**Figure 2:**
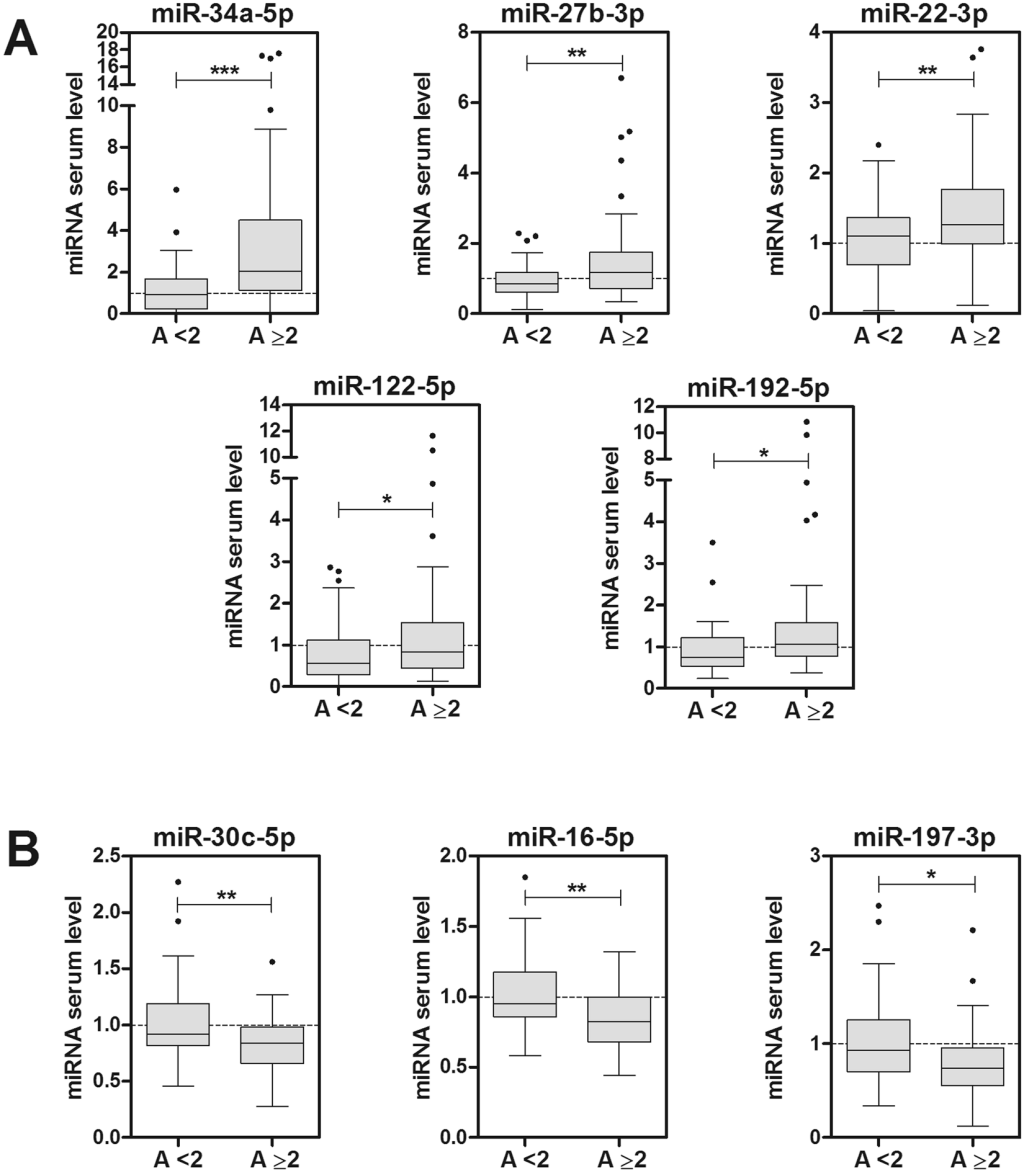
Serum miRNAs significantly altered in NAFLD patients with high SAF Activity. The study cohort was divided in two groups according to the SAF Activity scoring system. Fifty NAFLD patients were classified with Activity ≥ 2, defining Activity as the addition of grades of ballooning (from 0 to 2) and lobular inflammation (from 0 to 2). The level of each individual miRNA was normalized with the geometric mean of miR-25 and miR-15a. Five miRNA were found increased (**A**) and three decreased (**B**) in patients with A ≥ 2. Data represent fold-change of the normalized serum miRNA level. *p < 0.05, **p < 0.01, ***p < 0.001, t-test.

We analysed differences in the serum concentration of miRNAs between NAFLD patients with more severe and less severe disease. First, we grouped subjects following the SAF scoring system. NAFLD patients with significant disease are those with Activity ≥ 2, defining Activity as the addition of grades of ballooning (from 0 to 2) and lobular inflammation (from 0 to 2)^7,8^. Only 8 of the 16 miRNAs analysed showed significantly different serum levels in NAFLD patients with Activity ≥ 2. Five of them were induced (-34a, -27b, -22, -122 and -192) (Fig. 2A) and three were repressed (-30c, -16 and -197) (Fig. 2B).

**Figure 3:**
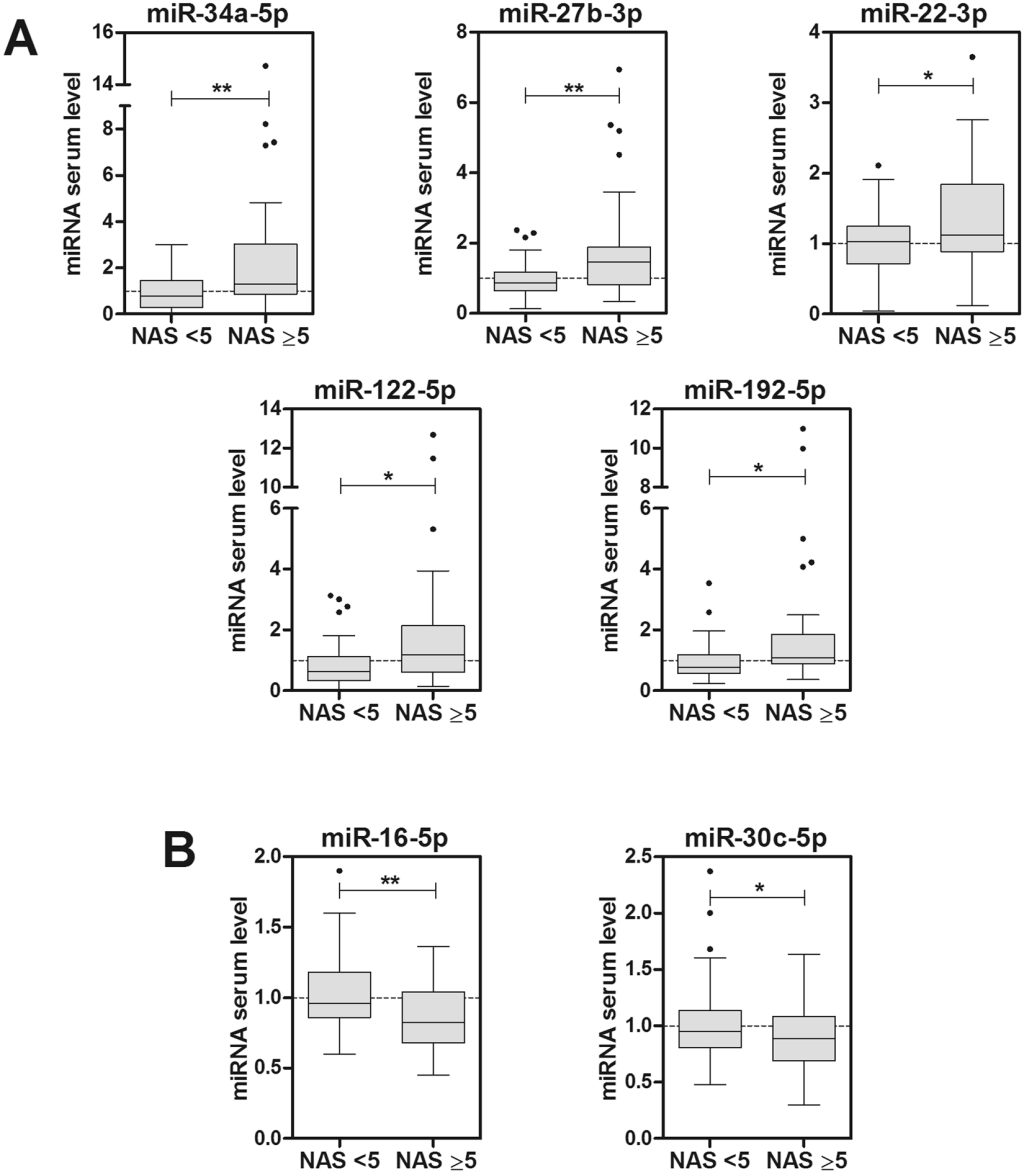
Serum miRNAs significantly altered in NAFLD patients with high NAS score. The study cohort was divided in two groups according to the NAS scoring system. Thirty-eight NAFLD patients were classified with NAS ≥ 5. The level of each individual miRNA was normalized with the geometric mean of miR-25 and miR-15a. Five miRNA were found increased (**A**) and two decreased (**B**) in patients with NAS ≥ 5. Data represent fold-change of normalized serum miRNA level. *p < 0.05, **p < 0.01, t-test.

We also assessed severity by the NAS system^5,6^, where the NAS score results from the addition of grades of steatosis (0–3), lobular inflammation (0–3) and hepatocellular ballooning (0–2). Patients with definite NASH are those with a NAS score ≥ 5. Results demonstrate that 7 of the 16 miRNAs were altered in the serum of patients with NAS ≥ 5. Four of the induced miRNAs were the same as those found in the SAF Activity ≥ 2 group (-34a, -27b, -122 and -192). In addition, we observed that the levels of miR-22 were also induced in patients with NAS ≥ 5 (Fig. 3A). Regarding repressed miRNAs, we observed a significant decrease in -16 and -30c in this group of patients (Fig. 3B).

**Figure 4:**
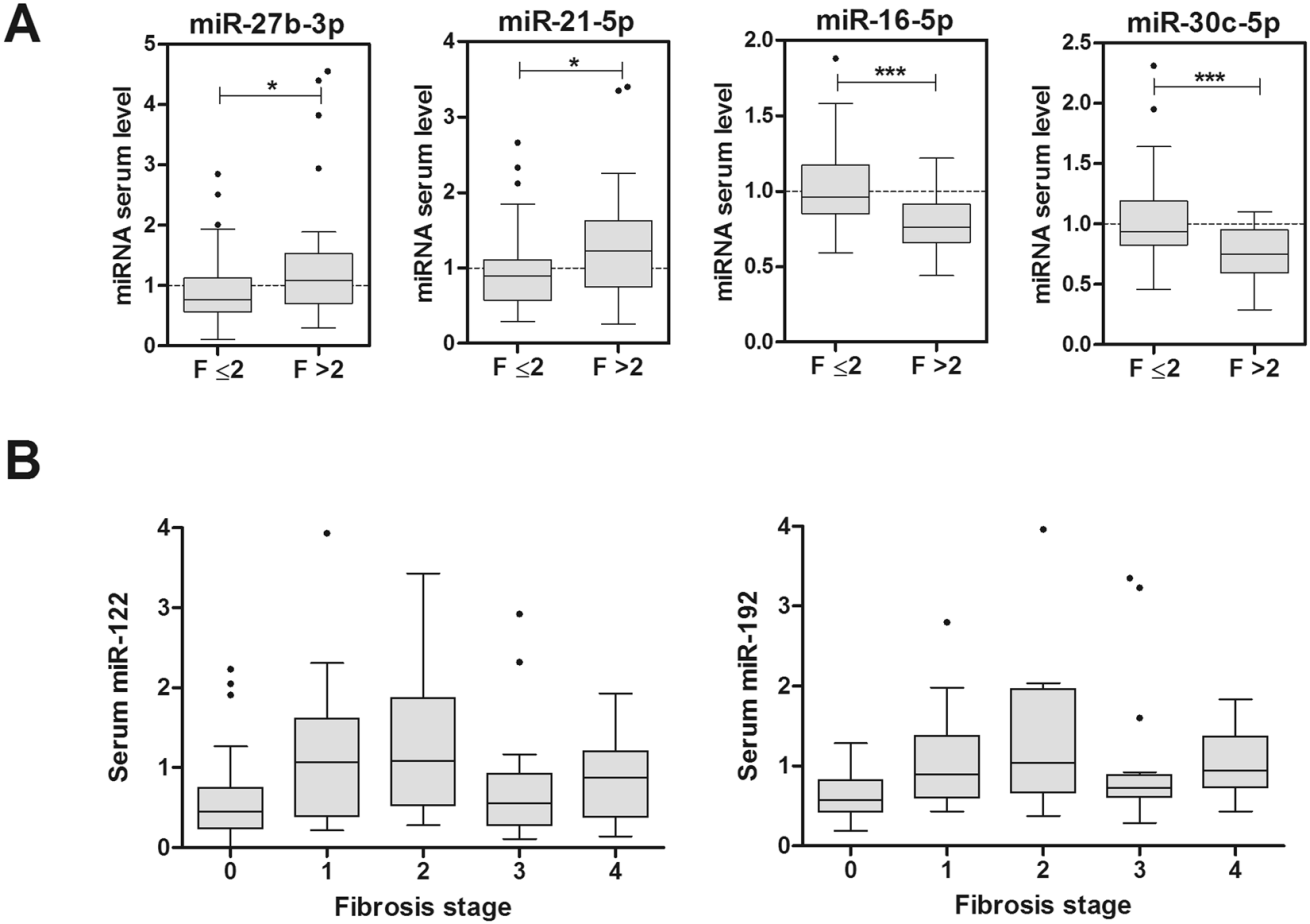
Changes in serum miRNAs according to the fibrosis stage in NAFLD patients. The study cohort was divided in either 2 (**A**) or 5 (**B**) groups according to the Fibrosis stage. Twenty-nine NAFLD patients were classified with Fibrosis stage >2 (F3 + F4). The level of each individual miRNA was normalized with the geometric mean of miR-25 and miR-15a. Data represent fold-change of normalized serum miRNA level. *p < 0.05, **p < 0.01, t-test.

Regarding fibrosis severity, we searched for miRNAs deregulated in patients with F > 2 (F3 & F4) and found that 4 miRNAs had a significant different serum level in these patients. Once again, -27b was induced and -16 and -30c were repressed. Moreover, in severe fibrosis patients, serum miR-21 levels were also increased (Fig. 4A). Some miRNAs, such as -122 and -192, that were significantly increased in patients with high SAF Activity or NAS score, were not induced in severe fibrosis. Results in Fig. 4B demonstrate that while these miRNAs tend to increase in mild fibrosis stages (F1 & F2) they drop to lower levels in severe fibrosis (F3 & F4). These dual behaviour prevents from significant differences between F0–2 and F3–4 groups.

In summary, from the 16 miRNAs previously postulated as biomarkers for NAFLD diagnosis and severity, we confirm that -27b, -34a, -22, -122, -192 and -21 are induced and -30c, -16 and -197 are repressed in the serum of severely diseased NAFLD patients. However, only three of them (-27b, -16 and -30c) were found consistently altered in both NASH and severe fibrosis.

### Association of circulating miRNAs with clinical variables

Serum miRNAs with increased levels in more severe NAFLD patients (-27b, -34a, -22, -122, -192 and -21) showed strong correlations with AST, ALT, ferritin levels and the APRI fibrosis score (APRI = AST to Platelet Ratio Index) (Supplementary Table 2). We also observed significant correlations between the miRNAs altered in NAFLD patients with severe fibrosis and the fibrosis test scores. The upregulated miR-27b correlated with FIB-4 and APRI, whereas downregulated -30c and -16 correlated with FIB-4, BARD and the NAFLD Fibrosis Score (Supplementary Table 2).

Interestingly, miR-16 also correlated with alkaline phosphatase (ALP) (R_Pearson_ = −0.28, p = 0.01), miR-192 with prothrombin (R_Pearson_ = 0.30, p = 0.004) and miR-122 with total bilirubin levels (R_Pearson_ = 0.30, p = 0.003).

### Diagnostic performance of circulating miRNAs

Previous studies have estimated the diagnostic performance of serum miRNAs by ROC curve analysis and associated parameters (AUROC, sensitivity, specificity, PLR, NLR, etc.) (see Table 1). We have also analysed the diagnostic performance of the differentially expressed miRNAs in our patient’s cohort to confirm or not their diagnostic value. We reasoned that because some miRNAs were induced and others repressed in severe NAFLD patients, the ratios between induced and repressed miRNAs could generate novel variables with increased diagnostic performance. Conventional serum-based markers and fibrosis indices were also analysed, and those performing better were compared with the miRNA biomarkers. Optimal cut-off values were determined using Youden’s index.

We analysed first the predictive value of miRNAs for patients with SAF Activity ≥ 2. The best performing miRNA was miR-34a, with a modest AUROC of 0.76. However, the miRNA ratios miR-34/197 and miR-192/197 showed better AUROCs of 0.81 and 0.78 respectively. The best performing conventional clinical marker was AST with an AUROC of 0.75. The sensitivity was similar for miRNA ratios and for AST (73–74% vs 74% for AST) but the specificity was higher for miRNA ratios (83–79% vs 64% for AST) (Table 3 & Supplementary Figure 1A).

**Table 3:** Performance of predictors of NASH and advanced fibrosis assessed by ROC curve analysis.

	SAF-Activity ≥2			NAS ≥5			Fibrosis >2		
	miR-34a/197	miR-192/197	AST	miR-192/30c	miR-27b/30c	AST	miR-27b/197	miR-27b/30c	FIB-4
AUC	0.81 (0.72–0.90)*	0.78 (0.69–0.88)	0.75 (0.65–0.85)	0.78 (0.69–0.88)	0.79 (0.69–0.88)	0.75 (0.64–0.85)	0.75 (0.65–0.86)	0.77 (0.67–0.87)	0.85 (0.76–0.93)
Optimal cut-off	4.43	2.55	33	1.62	2.83	33	2.28	2.34	1.68
Sensitivity	0.73 (0.58–0.85)	0.74 (0.60–0.85)	0.74 (0.59–0.85)	0.87 (0.72–0.96)	0.55 (0.38–0.71)	0.79 (0.63–0.90)	0.83 (0.64–0.94)	0.72 (0.53–0.87)	0.72 (0.53–0.87)
Specificity	0.83 (0.67–0.93)	0.79 (0.63–0.90)	0.64 (0.48–0.78)	0.69 (0.54–0.80)	0.89 (0.77–0.96)	0.59 (0.45–0.73)	0.60 (0.47–0.72)	0.74 (0.62–0.85)	0.86 (0.75–0.93)
PPV (precision)	0.83 (0.69–0.91)	0.80 (0.66–0.90)	0.71 (0.56–0.84)	0.66 (0.52–0.86)	0.78 (0.60–0.88)	0.58 (0.43–0.76)	0.49 (0.36–0.76)	0.57 (0.42–0.77)	0.70 (0.53–0.86)
NPV	0.72 (0.57–0.87)	0.72 (0.57–0.86)	0.67 (0.52–0.81)	0.88 (0.74–0.93)	0.74 (0.59–0.89)	0.80 (0.64–0.88)	0.88 (0.74–0.93)	0.86 (0.71–0.91)	0.87 (0.74–0.94)
PLR	4.17 (2.08–8.35)	3.45 (1.89–6.31)	2.07 (1.34–3.21)	2.76 (1.83–4.17)	4.97 (2.22–11.2)	1.92 (1.35–2.78)	2.09 (1.47–2.95)	2.85 (1.77–4.60)	5.07 (2.63–9.66)
NLR	0.33 (0.20–0.53)	0.33 (0.20–0.54)	0.40 (0.24–0.68)	0.19 (0.08–0.44)	0.50 (0.35–0.73)	0.35 (0.19–0.68)	0.29 (0.13–0.65)	0.37 (0.20–0.68)	0.32 (0.18–0.59)
Accuracy	0.77 (0.67–0.85)	0.74 (0.64–0.83)	0.70 (0.59–0.79)	0.74 (0.64–0.82)	0.73 (0.63–0.82)	0.67 (0.57–0.77)	0.66 (0.56–0.76)	0.71 (0.61–0.81)	0.82 (0.72–0.89)

^*^Values within parenthesis indicate confidence intervals.

AUROC, area under the curve of the receiver operating characteristic; PPV, positive predictive value; NPV, negative predictive value; PLR, positive likelihood rate; NLR, negative likelihood ratio.

Regarding the prediction of patients with NAS ≥ 5 we observed that miRNAs performed better than classical clinical markers. The best individual miRNA was miR-27b with an AUROC of 0.73; but the miRNA ratios miR-192/30c and miR-27b/30c performed better (AUROC = 0.78 and 0.79 respectively). The best classical clinical marker was again AST with a lower AUROC of 0.75. The best sensitivity was found for miR-192/30c (87% vs 79% for AST) and the best specificity was for miR-27b/30c (89% vs 59% for AST) (Table 3 & Supplementary Figure 1B).

Therefore, our results support that miR-34a, -192, -27b, and their ratios with -197 and -30c, are the most useful miRNA biomarkers to discriminate between NAFLD and NASH patients.

Finally, we evaluated the prediction of severe fibrosis in NAFLD patients. Results demonstrated that, in general terms, miRNAs do not show a clear advantage over conventional fibrosis algorithms. The best individual miRNA was -30c (downregulated in F > 2) with an AUROC of 0.72. Some miRNA ratios such as -27b/30c and -27b/197 performed slightly better (AUROC = 0.77 and 0.75 respectively). Nonetheless, FIB-4, with an AUROC of 0.85, outperformed miRNAs. Moreover, FIB-4, in agreement with previous studies^10^, had a high specificity (86%), indicating a good performance to rule out advanced fibrosis (Table 3 & Supplementary Figure 1C). Interestingly, the miRNA ratio -27b/197 showed a higher sensitivity (83% vs 72% for FIB-4) and could complement conventional fibrosis algorithms.

### Multivariate classification models based on serum miRNAs vs serum-based clinical variables

We also developed Multivariate PLS-DA (partial least squares regression – discriminant analysis) models to test if they are able to better discriminate patients according to their class membership, i.e. SAF Activity < 2 vs ≥ 2, NAS < 5 vs ≥ 5 or Fibrosis ≤ 2 vs > 2. Moreover, this approach allowed us to apply a robust cross-validation method. Table 4 and Supplementary Figure 2 summarize cross-validated results for the estimation of the generalizability of the three considered models using either clinical variables, miRNAs or both types of variables together.

**Table 4:** Assessment by leave-one-out cross validation (LOO-CV) of the predictive performance of PLS-DA models based on conventional serum-based clinical markers and/or miRNAs.

		Clinical	miRNAs	Clinical & miRNAs
SAF Act ≥2	AUROC	0.75	0.79	0.82
	Sensitivity	68.0 (53.3–80.5)^*^	74.0 (59.7–85.4)	66.0 (51.2–78.8)
	Specificity	78.6 (63.2–89.7)	71.4 (55.4–84.3)	78.6 (63.2–89.7)
	PLR	3.2 (1.7–5.8)	2.6 (1.6–4.3)	3.1 (1.7–5.7)
	NLR	0.4 (0.3–0.6)	0.4 (0.2–0.6)	0.4 (0.3–0.7)
	Variables	16	33	49
	LVs	2	3	2
NAS ≥5	AUROC	0.67	0.74	0.78
	Sensitivity	44.7 (28.6–61.7)	50.0 (33.4–66.6)	47.4 (31.0–64.2)
	Specificity	79.6 (66.5–89.4)	85.2 (72.9–93.4)	87.0 (75.1–94.6)
	PLR	2.2 (1.2–4.1)	3.4 (1.6–6.9)	3.6 (1.7–7.9)
	NLR	0.7 (0.5–0.9)	0.6 (0.4–0.8)	0.6 (0.4–0.8)
	Variables	16	33	49
	LVs	2	2	1
Fibrosis >2	AUROC	0.75	0.81	0.83
	Sensitivity	72.4 (52.8–87.3)	69.0 (49.2–84.7)	72.4 (52.8–87.3)
	Specificity	74.6 (62.1–84.7)	76.2 (63.2–86.0)	82.5 (70.9–91.0)
	PLR	2.9 (1.8–4.6)	2.9 (1.7–4.8)	4.15 (2.3–7.4)
	NLR	0.4 (0.2–0.7)	0.4 (0.2–0.7)	0.3 (0.2–0.6)
	Variables	16	33	49
	LVs	4	3	2

*Values within parenthesis indicate confidence intervals.

AUROC, area under the curve of the receiver operating characteristic; PPV, positive predictive value; NPV, negative predictive value; PLR, positive likelihood rate; NLR, negative likelihood rate.

Cross-validated AUROC values for clinical variables varied in the 0.67–0.75 range (Table 4), suggesting poor generalizable performances for these variables in NAFLD severity prediction. AUROC values increased when miRNAs and miRNA ratios where tested (0.74–0.81) and reached even higher values (0.78–0.83) when all variable (clinical + miRNAs) where considered together (Table 4).

Thus, the cross-validated PLS-DA models developed using the set of microRNAs were able to classify patients better than those based on conventional serum clinical variables, for the three severity groups: high SAF Activity, high NAS score and high fibrosis stage. However, the best classification was achieved when both clinical and miRNA variables are combined in the same model. Results suggest that incorporation of serum miRNAs to current or future predictive algorithms could significantly improve the non-invasive diagnose of NAFLD patients at risk.

### Are the miRNA biomarkers specific for NAFLD severity?

The serum miRNAs associated to NASH and advanced fibrosis in this study could merely be general biomarkers of liver disease progression. It would be then desirable to test if these miRNAs are also associated to other pathological conditions such as alcohol- or drug-induced liver injury, viral hepatitis, etc. To test this possibility, we have also analysed the levels these miRNA biomarkers in the sera of a small group of patients with drug-induced liver injury (DILI).

DILI patients showed significantly higher levels of liver enzymes (AST, ALT, ɣ-GT and ALP), bilirubin, triglycerides and platelets, while serum albumin levels were found decreased (Table 5). The causative agents likely responsible for DILI in these patients belonged to the therapeutic groups of antibiotics (e.g. amoxicillin/clavulanate, ceftriaxone), NSAID-painkillers (e.g. metamizole, naproxen), chemotherapeutics (e.g. capecitabine, methotrexate), statins (e.g. atorvastatine) and anabolic androgenic steroids (e.g. havoc/epistane).

**Table 5:** Baseline characteristics of the DILI study cohort.

	NL (n = 17)	DILI (n = 17)	
Age (years)	42.9 ± 9.3	53.3 ± 19.3	
Sex			
Male	7 (41.2%)	11 (64.7%)	
Female	10 (58.8%)	6 (35.3%)	
Body mass index (kg/m^2^)	26.2 ± 3.6	24.6 ± 2.7	
Glucose (mg/dL)	89.9 ± 10.7	91.6 ± 14.2	
Triglycerides (mg/dL)	106.7 ± 36.3	211.9 ± 138.2	**
Total cholesterol (mg/dL)	203.7 ± 37.4	188.9 ± 76.9	
Total bilirubin (mg/dL)	0.6 ± 0.2	11.2 ± 15.4	*
Albumin (g/dL)	4.5 ± 0.5	3.8 ± 0.5	***
Platelets (10^3/µL)	245.2 ± 75.1	341 ± 125	*
ALT (IU/L)	29.8 ± 16.7	94.6 ± 48.7	***
AST (IU/L)	26.0 ± 8.8	72.7 ± 39.8	***
ɣ-GT (IU/L)	50.8 ± 51.4	347.8 ± 275.2	***
ALP (IU/L)	86.1 ± 32.7	320.6 ± 184.9	***
Hb (g/dL)	13.9 ± 0.9	13.1 ± 1.9	

Data are shown as mean ± standard deviation. NL, normal liver; DILI, drug-induced liver injury; ALT, alanine aminotransferase; AST, aspartate aminotransferase; ɣ-GT, gamma-glutamyltransferase; ALP, alkaline phosphatase; Hb, Hemoglobin. *p < *0.05*, **p < *0.01*, ***p < *0.001* (t-test).

**Figure 5:**
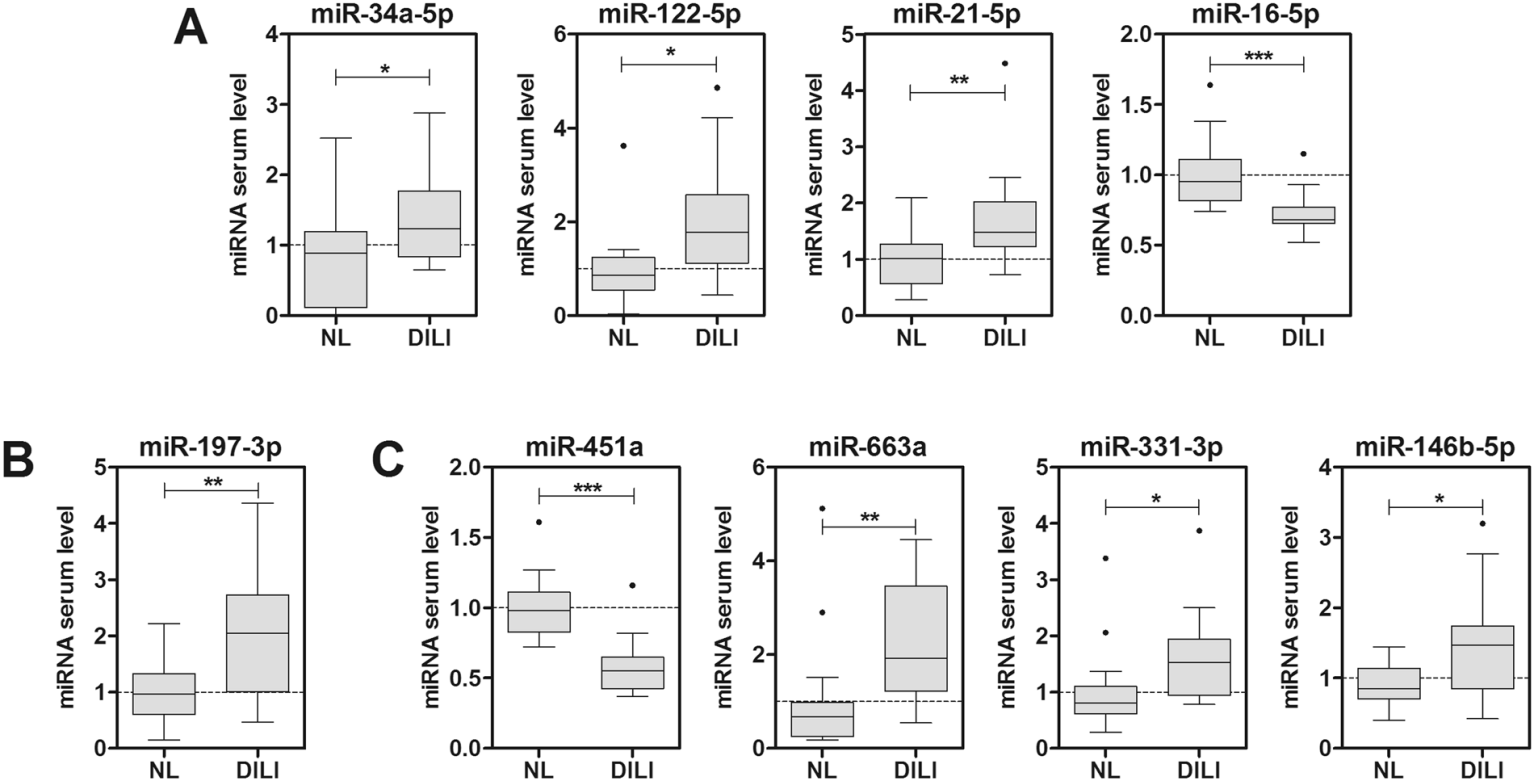
Serum miRNA profile in DILI patients. RNA was purified from sera of normal liver subjects (NL, n = 17) and drug-induced liver injury patients (DILI, n = 17). The level of each individual miRNA was normalized with the geometric mean of miR-25 and miR-15a. Data represent fold-change of normalized serum miRNA level. *p < 0.05, **p < 0.01, t-test.

Analysis of the serum miRNA validated as biomarkers for NAFLD severity revealed that 34a, -122, -21 and -16 were also altered in DILI (Fig. 5A), whereas -27b, -192, -22 and -30c did not change in DILI (data not shown). Thus, it is tempting to speculate that these latter four miRNAs could be classified as specific for NAFLD severity, though this needs to be demonstrated in larger cohorts of patients with DILI and other liver diseases. Another interesting miRNA with a very distinct profile between severe NAFLD patients and DILI patients was -197, that showed lower serum levels in NASH (Fig. 2B), but induced levels in DILI (Fig. 5B).

Finally, other miRNAs, that were not associated to NAFLD severity in this study (-451, -663a, -146b and -331) were found significantly altered in patients with hepatotoxicity (Fig. 5C). These miRNAs could be classified as DILI-specific.

### Are the miRNA biomarkers for NAFLD severity identified so far the best miRNA candidates?

Among the 14 previous studies aimed at finding miRNA biomarkers for NAFLD only 3 performed non-biased screenings (PCR-based arrays or next-generation sequencing)^12,16,18^. Most others focused on particular miRNAs based on previous knowledge and, consequently, did not perform *de novo* screenings. Therefore, it is possible that some good miRNA biomarkers have been overlooked. To test this possibility, we performed a pilot RNAseq analysis in the sera of a small group of NAFL (n = 4) and NASH (n = 4) patients.

Only 178 mature miRNAs could be confidently detected in > 50% of patients, which represents ca. 7% of all mature human miRNAs in the miRBase. Results confirm the low sensitivity of RNAseq in serum when compared with conventional qPCR arrays, which have shown to be able to detect more than 25% of the miRNAs^18^. Among the 7 miRNAs associated to NASH (Figs 2 & 3) 5 were confirmed by RNAseq as differentially expressed in the serum of NASH patients, whereas 2 of them (-27b and 34a) were not confidently detected. In addition, 39 new miRNAs were found significantly altered in NASH (25 increased and 14 decreased) (Supplementary Table 3). These miRNAs have not been reported as associated to NAFLD severity before and, therefore, it is tempting to suggest that the research in this field is still open. Among the new miRNA identified we found -125b, -193a/b, -320b/c/e, -378a/c/g and -483 with significant inductions; and -let7a/d/f, -27a, -93, -150, -215 and -223 with important repressions. The relevance of these new miRNAs as circulating biomarkers for NASH needs to be confirmed and validate in suitable cohorts of patients, whereby they may become circulating biomarkers with better prediction performance than those identified and validated so far.

## Discusion

Sixteen of the 18 serum miRNAs previously described as associated with NAFLD were confidently detected in our study cohort, but only 9 of them (-34a, -192, -27b, -122, -22, -21, -197, -30c and -16) appeared significantly altered in more severe NAFLD patients.

It is important to remark that three of the previous studies pursued to identify miRNAs associated with the NAFLD condition rather than with NAFLD severity^16–18^ (Table 1). Two of them included cross-sectional analysis, where liver biopsy was not indicated and severity could not be graded. Yamada *et al*.^17^ analysed selected serum miRNAs in patients diagnosed with NAFLD by ultrasound scan. Authors found that miR-34a and miR-122 (among others) were higher in the serum of NAFLD participants. Zarrinpar *et al*.^18^ searched for miRNAs with different expression in tweens showing discordance in NAFLD. Liver MIR was used for NAFLD diagnosis. One of the most significant miRNAs identified, -30c, was reduced in both concordant and discordant tweens with NAFLD. In a third study, patients were biopsied but the performance of miRNAs to predict NASH or fibrosis was not evaluated^16^; authors identified a serum miRNA panel (including -122, 192, and -27b) with high accuracy in predicting NAFLD patients. Our study and others^12,19,20,22,23,27^ demonstrate that some of these miRNAs (i.e. -34a, -122 and -192) do not only associate with the NAFLD condition but also with disease severity. Moreover, we have proven for the first time that serum -27b and -30c are significantly altered in both NASH and advanced fibrosis, which makes these miRNAs useful biomarker for NAFLD patients at risk.

Nonetheless, diagnosis of the NAFLD condition in the general population is routinely assessed by ultrasonography or other imaging methods^3^, whereas no highly sensitive and specific tests are available to differentiate NASH/fibrosis from simple steatosis^10^. Therefore, most of the studies on circulating miRNA and NAFLD published so far have intended to identify miRNA biomarkers associated with NAFLD severity (Table 1).

Multiple studies have shown that -122 is significantly increased in the serum of NASH patients^12,19,20,22,23,27^. However, there are fewer previous studies analysing serum -122 in advanced fibrosis in NAFLD. One of them showed increased -122 serum levels^12^, whereas other supported the opposite conclusion (i.e. decreased -122 serum levels in severe fibrosis)^26^. A potential explanation is that serum -122 has a dual behaviour: increases in mild fibrosis and decreases in the most severe stages, as demonstrated by Akuta *et al*.^13^. Our results agree with this dual behaviour and extend it to other interesting miRNAs such as -192. This is a relevant finding as the differences among the fibrosis stages are cancelled when F0–2 and F3–4 are compared, which limits the use of -122 and other miRNA with similar behaviour in the prediction of severe fibrosis in NAFLD. Therefore, -122 could be postulated as a biomarker for NASH, but it does not seem useful as a biomarker for advanced fibrosis. miR-122 represents one of the most abundant miRNAs in the liver, where it plays a role in cholesterol and free fatty acid metabolism, hepatocellular carcinoma growth, and hepatitis C virus replication^30^. Circulating miR-122 correlates with the extent of hepatic cell death after viral-, alcohol-, chemical- and drug-induced liver injury, thus suggesting that serum miR-122 levels purely indicate hepatic cell death in different disease conditions^31^. This point of view is in agreement with the strong correlation between -122 and liver transaminases AST and ALT observed in this study, as well as with the increase observed in DILI patients. Similarly, the similar alteration of -34a, -21 and -16 observed in severe NAFLD and DILI could indicate these miRNAs also behave as universal biomarkers for hepatic injury.

The serum levels of miR-192^12,20,23^, miR-34a^22,23^ and miR-22^24,32^ have also been found increased in NASH patients, and our results fully agree with these findings. Similarly, serum miR-197 was shown to negatively correlate with liver inflammation in NAFLD^21^, which is also in concordance with our results of lower serum -197 in NASH patients. Thus, our study validates previous reports showing serum -122, -34a, -192, -22 and -197 to be significantly altered in NASH patients.

Our results regarding serum -16 show discrepancies with two previous studies. Cermelli *et al*.^22^ found -16 to be increased in NAFLD patients with simple steatosis (not in NASH), whereas Liu *et al*.^23^ showed that serum -16 increased in NASH patients and correlated with fibrosis stage. On the contrary, we have shown significant decreases in -16 levels both in NASH and in advanced fibrosis, and a significant negative correlation of this miRNA with AST and the fibrosis prediction scores. A possible explanation is that -16 is also abundant in non-liver cells such as erythrocytes^33^, and can therefore be significantly influenced by liver-unrelated processes (e.g. haemolysis). Thus, the usefulness of this miRNA still needs to be confirmed and, if there is a choice, it would be advisable to select miRNA biomarkers with substantial liver-specific and/or high hepatic expression. In this regard, an important finding of this study is the observed fair relationship between liver and serum levels for most of the validated miRNAs. Moreover, results by Di Mauro *et al*.^34^ confirm that several of these key miRNAs (i.e. -192, -27b, -197 and -30c) are altered in the extracellular medium of human hepatic cells upon incubation with fatty acids. The alterations observed in the culture media are in the same direction as in human serum (i.e. -192 and -27b increased whereas -197 and -30c decreased), which reinforces the relevance of the miRNAs validated in this study.

Regarding the diagnosis value of these miRNAs, we have observed similar classification performances of miRNAs vs. conventional serum markers, such as transaminases. However, we also demonstrate that the ratios between induced and repressed miRNAs perform better than serum markers in NASH prediction. In previous studies, the best postulated miRNAs for NASH prediction were: -34a, -122, -192 and -375^12,19,22,23^. ROC curve analysis with these miRNAs demonstrated AUROC in the range 0.68–0.76. Moreover, a panel based on -122, -192 and -21 showed an AUROC of 0.81^20^ (Table 1). Our results are in agreement, as the best performing miRNA for high SAF Activity (-34a) and for high NAS score (-27b) showed AUROC of 0.76 and 0.73 respectively. Interestingly, these values increased substantially when miRNA ratios were used (AUROC = 0.81–0.79). Moreover, their diagnosis performance was superior to that of AST (AUROC = 0.75).

The potential of miRNAs for predicting advanced fibrosis in NAFLD patients has not been sufficiently addressed. As stated above, two studies, with contradictory results, have postulated a predictive role for decreased (AUROC = 0.82)^26^ and increased (AUROC = 0.61)^12^ -122 in severe fibrosis. Conversely, our results and others^13^ demonstrate a dual behaviour for -122 in mild vs severe fibrosis that prevents its use in fibrosis prediction. In another study, a predictive role for increased -16 (AUROC = 0.71)^23^ was proposed, but we have found decreased -16 in F3–4, rather than increased levels. Moreover, we have shown for the first time an association of -27b, -21 and -30c with F3–4.

Regarding the performance in severe fibrosis diagnosis, the best individual miRNA was miR-30c (AUROC = 0.72) and the best ratio was -27b/30c (AUROC = 0.77). These values are similar to those achieved by some fibrosis algorithms such as APRI and BARD (both with AUROC = 0.76). However, the popular index FIB-4 showed better performance (AUROC = 0.85). Intriguingly, miR-27b/197 demonstrated a higher sensitivity (83% vs 72% for FIB-4), indicating that this miRNA ratio could perform better in classifying severe fibrosis patients in the right group. Current fibrosis algorithms^31^ do not include any circulating miRNA and we hypothesized that miRNAs could improve their predictive performance.

Finally, we have developed predictive models based on PLS-DA followed by LOO-CV to estimate their generalizable performance. They were built with serum-based clinical biomarkers, with serum miRNAs and with both types of variables together. Multivariate modelling provided a good fit between the data and the calibration models, as shown by the non-cross-validated AUROC values depicted in Supplementary Figure 2. However, these estimates can be subject to an optimistic bias compared to the results obtained with future unknown samples. Indeed, AUROC values after cross-validation were lower. There is no AUROC threshold value that indicates a good discrimination between classes. Nevertheless, if we take into account that a diagnostic tool is good if the AUROC is > 0.8 and excellent if the AUROC is > 0.9^35^, the cross validated AUROC of 0.7–0.8 observed in the clinical and miRNA prediction models suggest poor performance of both types of serum-based biomarkers, though miRNA functioned always slightly better. A novel approach would be to combine both types of serum-based biomarkers. Our preliminary results support such an approach as they demonstrate that combined models outperformed always the models based only on clinical or miRNA biomarkers, and reached cross-validated AUROC of 0.82 and 0.83 for SAF Activity and severe fibrosis, respectively.

We conclude that: 1) Even though 14 studies on predictive serum miRNAs in NAFLD have postulated 18 different miRNAs, only 5 of them appear in more than one study. 2) The validation of these results in an independent cohort has confirmed only 9 out of the 18 miRNAs as associated with NAFLD severity (-34a, -192, -27b, -122, -22, -21, -197, -30c and -16), being 5 of them NAFLD-specific as compared with DILI patients. 3) Some miRNAs such as -192, -27b and -122 are abundantly expressed in liver and confidently detected in serum (Ct 29–31), whereas others such as -16 show strong discrepancies between liver and serum levels, which limits their rational use in NAFLD diagnosis. 4) miRNAs with increased levels in the sera of more severe patients (e.g. -192, -27b and -122) display strong correlation with serum transaminases. 5) The classification performances of validated miRNAs for patients with high SAF Activity or NAS score are better than those reached by conventional serum-based biomarkers, whereas for advanced fibrosis prediction, miRNAs do not demonstrate better performance than FIB-4, despite higher sensitivity. 6) Exploratory serum RNAseq analysis suggest the existence of additional miRNA biomarkers for NASH not investigated yet.

Our results support that the research in this field is still open and that the validated miRNAs of this study can become useful biomarkers for the diagnosis of NAFLD patients at risk, especially by complementing and improving current or future prediction algorithms.

## Methods

### Patients and control subjects

This study comprised 75 biopsy-proven NAFLD patients (25 NAFL and 50 NASH) and 17 non-NAFLD controls who underwent laparoscopic cholecystectomy (see baseline characteristics in Table 2). NAFLD patients and non-NAFLD controls drank less than 20 g/day of alcohol, and other potential causes of liver disease (viral, autoimmune, hepatotoxic drugs, iron overload, Wilson’s disease, etc.) were excluded. Moreover, non-NAFLD control subjects had normal liver by laparoscopic analysis, and normal levels of fasting glucose, cholesterol, TG and liver enzymes.

NAFLD was diagnosed by percutaneous liver biopsy and ultrasonography. Hematoxylin-eosin and Masson´s trichrome-stained paraffin-embedded liver biopsy sections were examined and interpreted by the same experienced hepatopathologist (J. P-R), who was unaware of the clinical data. All liver biopsies showed more than 10 complete portal tracts. Steatosis was assessed as outlined by Brunt *et al*.^5^ grading the percentage of steatotic hepatocytes in: grade 0, <5%; grade 1, 5–33%; grade 2, >33–66%; and grade 3, >66%. Minimal criteria for the histological diagnosis of NASH included the combined presence of grade 1 steatosis, hepatocellular injury (ballooning) and lobular inflammation, with or without fibrosis. Disease severity was diagnosed according to both NAS^5,6^ and SAF^7,8^ scoring systems. Fibrosis staging was simplified into four categories: 1, centrilobular/perisinusoidal; 2, centrilobular plus periportal; 3, bridging fibrosis; and 4, cirrhosis. Anticoagulated venous blood was extracted between 8 and 10 am, after an overnight fasting, by the time of liver biopsy (in NAFLD patients) or just before cholecystectomy (in non-NAFLD controls). Blood was collected in siliconized tubes and centrifuged at 2500 × g for 10 min. Serum samples were stored at −80 °C.

This study also included 17 patients with drug-induced liver injury (DILI) (see baseline characteristics in Table 5). In these patients, alcohol history was insignificant and evaluation for viral hepatitis, autoimmune, genetic and metabolic liver diseases was negative. The absence of other causes of liver disease, the temporal relationship between onset of symptoms and the use of the drug, and the normalization after its withdrawal were all highly suggestive of DILI. According to the RUCAM classification (Roussel Uclaf Causality Assessment Method)^36^ the diagnosis of DILI was probable (RUCAM score between 7 and 8 points).

The study was conducted in accordance with the 1975 Declaration of Helsinki and with local and national laws, and was approved by the Human Ethics Committee of La Fe University Hospital in Valencia (n° 2013/0232 & 2012/0452). Written informed consent was obtained from all participants.

### Purification and quantification of miRNAs in serum

Total RNA was extracted from 300 µL of human serum using Trizol LS reagent (Invitrogen, Barcelona, Spain) followed by the miRNeasy Mini Kit (Qiagen, Madrid, Spain). Prior to serum RNA purification, we added 20 µg of RNase-free glycogen (Roche Applied Sciences, Barcelona, Spain) as a carrier. Purified RNA was reverse transcribed in two steps: polyadenylation with 1U Poly(A) polymerase from *E.coli* (New England BioLabs, Ipswich, USA) and reverse transcription with an universal anchor primer (CGACTCGATCCAGTCTCAGGGTCCGAGGTATTCGATCCTAACCCT CTCCTCGGTATCGAGTCGCACTTTTTTTTTTTTVN) and 200U M-MLV reverse transcriptase (Invitrogen, Barcelona, Spain)^37^.

Diluted cDNA was amplified in a LightCycler 480 Instrument (Roche Applied Science) using LightCycler 480 Probes Master (Roche Applied Science) and the appropriate primers: a universal reverse primer (CCAGTCTCAGGGTCCGAGGTATTC), a specific forward primer for each miRNA (Supplementary Table 1) and a universal TaqMan probe (FAM-TCTCCTCGGTATCGAGTCGCACT-TAMRA)^37^. The concentration of miRNAs in the serum was calculated with the 2−ddCt method^38^.

Sample to sample variations were normalized with the geometric mean of two miRNAs (miR-15a-5p and miR-25-5p), which are confidently expressed in human serum, and show low variability. Indeed, these two miRNAs showed the best stability scores of our dataset according to geNorm, an algorithm for evaluating and determining the most stable reference (housekeeping) genes from a gene expression dataset in a given sample panel^39^. Moreover, miR-15a and miR-25 have been previously used as reference stable miRNAs for normalization^40–45^.

### Serum miRNAs sequencing

sRNA cDNA libraries were prepared from total serum RNA from 8 patients (4 NAFL vs 4 NASH) from the study cohort (Table 2) using NEBNext® Multiplex Small RNA Library Prep Set (#E7300 y #7580; New England BioLabs®, Inc., Ipswich, MA, USA) and miRNA were eluted using Pippin Prep System (Sage Science, Inc., Beverly, MA, USA) selecting a range size of 120–200 pb. These libraries were sequenced in a NextSeq 500 System using a 150 cycles Kit (Illumina, Inc., San Diego, CA, USA). The serum samples had a median of 237554 miRNA reads (from 68364 to 512598 reads). Datasets and additional information are deposited in the Gene Expression Omnibus (GEO) Database (GSE114923).

### Data analysis and statistics

Qualitative variables are presented as absolute (number, n) and relative (percentage, %) frequencies. Quantitative variables are expressed as measures of central tendency (mean) and dispersion (standard deviation). To assess differences between two groups, qualitative variables were analysed by chi square-test, and quantitative variables by t test for normal distribution and equal variances, or Mann–Whitney U test for non-normal distribution or heteroscedastic variances. For more than two groups, the statistical significance of the mean differences was evaluated by one-way ANOVA and Tukey HSD *post hoc* test. Correlation between two variables was studied with the Pearson or Spearman test, depending on whether the variables had a normal (parametrical) distribution or not.

The accuracy of predictors of NASH and advanced fibrosis was investigated by determining the area under the receiver operating characteristic (ROC) curve (AUROC). The appropriate cut-off for the best sensitivity and specificity was assessed by Youden Index. Significance was set at a value of P < 0.05.

The interrelationship among multiple miRNAs was modelled by PLS-DA models to overcome issues related to multicollinearity among independent variables, and the relative large number of variables compared to the sample size. PLS-DA is an extensively used multivariate classification method that aims to model the linear relationship between a data matrix and a vector of responses (i.e. classes), extracting a set of latent variables (LVs) explaining the variation in the data correlated with the class vector^46^. The number of LVs retained determines the complexity of the model. Selecting a high or low number of LVs could lead to overfitting or underfitting, respectively and so, it must be carefully selected. Here, cross-validated figures of merit were used to select the optimum number of LVs and, using autoscaled data, the number of LVs providing the lowest error of classification, estimated by leave-one out-cross validation (LOO-CV), was selected. Likewise, LOO-CV was used for the evaluation of the generalization performance of the developed classifiers using the AUROC, sensitivity, specificity, positive and negative predictive values (PPV and NPV, respectively), the negative and positive likelihood rates (NLR and PLR, respectively) and the overall accuracy as performance estimates.

## Electronic supplementary material

Supplementary Information
