# Supplementary material for: Non-invasive prediction of NAFLD severity: a comprehensive, independent validation of previously postulated serum microRNA biomarkers
Source: Sci Rep. 2018 Jul 13;8:10606. doi: 10.1038/s41598-018-28854-4 (PMC6045608; doi:10.1038/s41598-018-28854-4)
Supplement: Supplementary file 1 — Supplementary Information [file 41598_2018_28854_MOESM1_ESM.pdf]

Supplementary information to:

Non-invasive prediction of NAFLD severity: a comprehensive, independent validation of previously postulated serum microRNA biomarkers.

Mireia López-Riera[1], Isabel Conde[1,2], Guillermo Quintas[1,3], Laia Pedrola Vidal[4], Ángela Zaragoza[2], Judith Perez-Rojas[5], Mario Salcedo[6], Salvador Benlloch[2], José V. Castell[1,7,8] and Ramiro Jover[1,7,8*]

Supplementary Tables

Supplementary Table 1: Specific forward primers for miRNA qRT-PCR

**miR-15a-5p**	AGCAGCACATAATGGTTTGT	**miR-16-5p**	GTAGCAGCACGTAAATATTG
**miR-25-5p**	GCATTGCACTTGTCTCGGT	**miR-27b-3p**	CAGTTCACAGTGGCTAAGTTCT
**miR-21-5p**	AGACTAGCTTATCAGACTGATG	**miR-29a-3p**	GCAGTAGCACCATCTGAAATC
**miR-22-3p**	GGCTGCCAGTTGAAGAACT	**miR-30c-5p**	AGTGTAAACATCCTACACTCTCA
**miR-34a-5p**	TCAGTGGCAGTGTCTTAGCT	**miR-122-5p**	ACAGTGGAGTGTGACAATG
**miR-146b-5p**	CCAGTGAGAACTGAATTCCATAG	**miR-181c-5p**	GACATTCAACCTGTCGGTGAG
**miR-192-5p**	CAGCTGACCTATGAATTGACAG	**miR-197-3p**	TTCACCACCTTCTCCACCCA
**miR-331-3p**	CCCCTGGGCCTATCCTAGA	**miR-375**	GTTCGTTCGGCTCGCGTGA
**miR-451a**	AGAAACCGTTACCATTACTGAGT	**miR-663a**	AGGGGCGCCGCGGGAC

Supplementary Table 2: Correlations between serum miRNA and clinical variables. miRNAs up-regulated in the serum of severe NAFLD patients strongly correlate with transaminases, ferritin and the APRI fibrosis score. However, miRNAs down-regulated correlate with FIB4, BARD and NAFLD Fibrosis score.

	**AST**	**ALT**	**Ferritin**	**APRI**	**FIB4**	**BARD**	**NAFLD_FS**
**miR-21-5p (r)**	0.30**	0.34**	0.19	0.30**	0.12	-0.05	0.03
**miR-22-3p (r)**	0.29**	0.26*	0.24*	0.29**	0.18	0.04	0.04
**miR-122 (r)**	0.67***	0.82***	0.33**	0.54***	0.07	-0.33**	-0.14
**miR-34a-5p (r)**	0.39***	0.24*	0.34**	0.36***	0.22*	0.17	0.11
**miR-192-5p (r)**	0.72***	0.84***	0.31**	0.62***	0.13	-0.29**	-0.06
**miR-27b-3p (r)**	0.66***	0.68***	0.35**	0.61***	0.25*	-0.15	0.02
**miR-30c-5p (r)**	-0.11	0.04	-0.01	-0.17	-0.29**	-0.24*	-0.34**
**miR-16-5p (r)**	-0.24*	-0.10	-0.08	-0.25*	-0.27**	-0.24*	-0.22*

(r) Pearson's correlation coefficient. (* $p<0.05$; ** $p<0.01$; *** $p<0.001$)

Supplementary Table 3: Differentially expressed miRNAs in the serum of NASH patients (n=4) vs NAFL patients (n=4) analysed by miRNAseq**:**

miRNA-ID	Fold (Log2)	Fold Change	Statistic (t)	p-value
hsa-mir-100-5p	1,84	3,59	4,30	0,002
hsa-mir-100-5p	1.8	3.6	4.3	0.0024
hsa-mir-125b-1-5p	1.4	2.7	2.9	0.0195
hsa-mir-125b-2-5p	2.4	5.2	4.4	0.0022
hsa-mir-193a-5p	2.8	6.8	5.8	0.0004
hsa-mir-193b-5p	4.9	30.0	7.7	0.0000
hsa-mir-210-3p	0.7	1.6	2.4	0.0434
hsa-mir-30a-5p	1.8	3.6	5.3	0.0007
hsa-mir-320b-1-3p	1.4	2.7	3.2	0.0122
hsa-mir-320b-1-5p	2.2	4.7	3.5	0.0076
hsa-mir-320b-2-3p	2.5	5.6	4.1	0.0030
hsa-mir-320c-1-3p	2.3	4.8	3.6	0.0064
hsa-mir-320c-2-5p	2.0	4.0	3.8	0.0047
hsa-mir-320e-5p	1.2	2.3	2.4	0.0397
hsa-mir-324-3p	0.9	1.9	2.8	0.0223
hsa-mir-378a-3p	1.9	3.8	3.7	0.0054
hsa-mir-378c-5p	2.3	5.0	3.2	0.0117
hsa-mir-378g-5p	1.3	2.5	2.8	0.0224
hsa-mir-483-5p	2.9	7.5	5.2	0.0007
hsa-mir-885-3p	1.3	2.4	2.4	0.0423
hsa-mir-1228-5p	1.7	3.2	3.4	0.0086
hsa-mir-1303-3p	0.9	1.9	2.8	0.0223
hsa-mir-1307-3p	0.9	1.8	2.8	0.0236
hsa-mir-1468-5p	0.8	1.7	2.4	0.0413
hsa-mir-3591-3p	2.9	7.5	4.5	0.0018
hsa-mir-6734-5p	1.3	2.4	2.6	0.0317
hsa-let-7a-1	-2.0	0.2	-4.2	0.0029
hsa-let-7d	-1.2	0.4	-2.5	0.0371
hsa-let-7f-2	-1.9	0.3	-2.8	0.0236
hsa-mir-27a-5p	-1.4	0.4	-4.2	0.0028
hsa-mir-92b-3p	-0.6	0.6	-2.4	0.0396

hsa-mir-93-5p	-1.1	0.5	-3.0	0.0164
hsa-mir-107-3p	-0.7	0.6	-2.4	0.0453
hsa-mir-150-3p	-0.7	0.6	-2.5	0.0374
hsa-mir-150-5p	-1.0	0.5	-4.3	0.0024
hsa-mir-215-5p	-1.4	0.4	-3.5	0.0073
hsa-mir-223-5p	-1.5	0.3	-2.4	0.0402
hsa-mir-330-5p	-0.7	0.6	-2.3	0.0474
hsa-mir-485-5p	-0.8	0.6	-2.5	0.0384
hsa-mir-582-3p	-1.5	0.4	-2.4	0.0423
**hsa-mir-122-5p**	2.9	7.7	4.2	0.0026
**hsa-mir-192-5p**	1.3	2.5	1.8	0.1021
**hsa-mir-22-3p**	1.8	3.5	3.2	0.0124
**hsa-mir-16-5p**	-1.6	0.3	-3.2	0.0121
**hsa-mir-30c-5p**	-0.6	0.7	-1.0	0.3252

Supplementary Figure 1

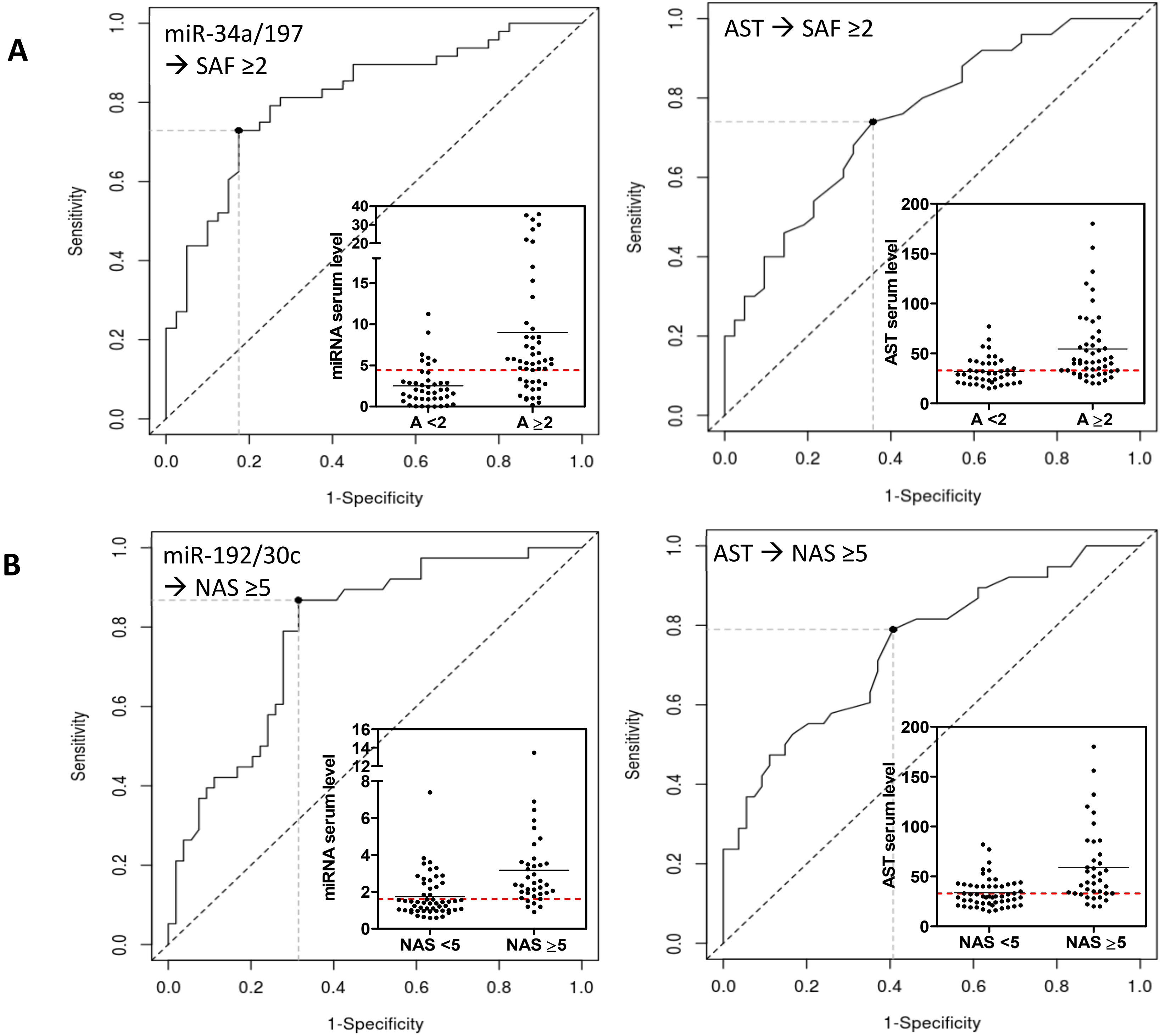

Supplementary Figure 1

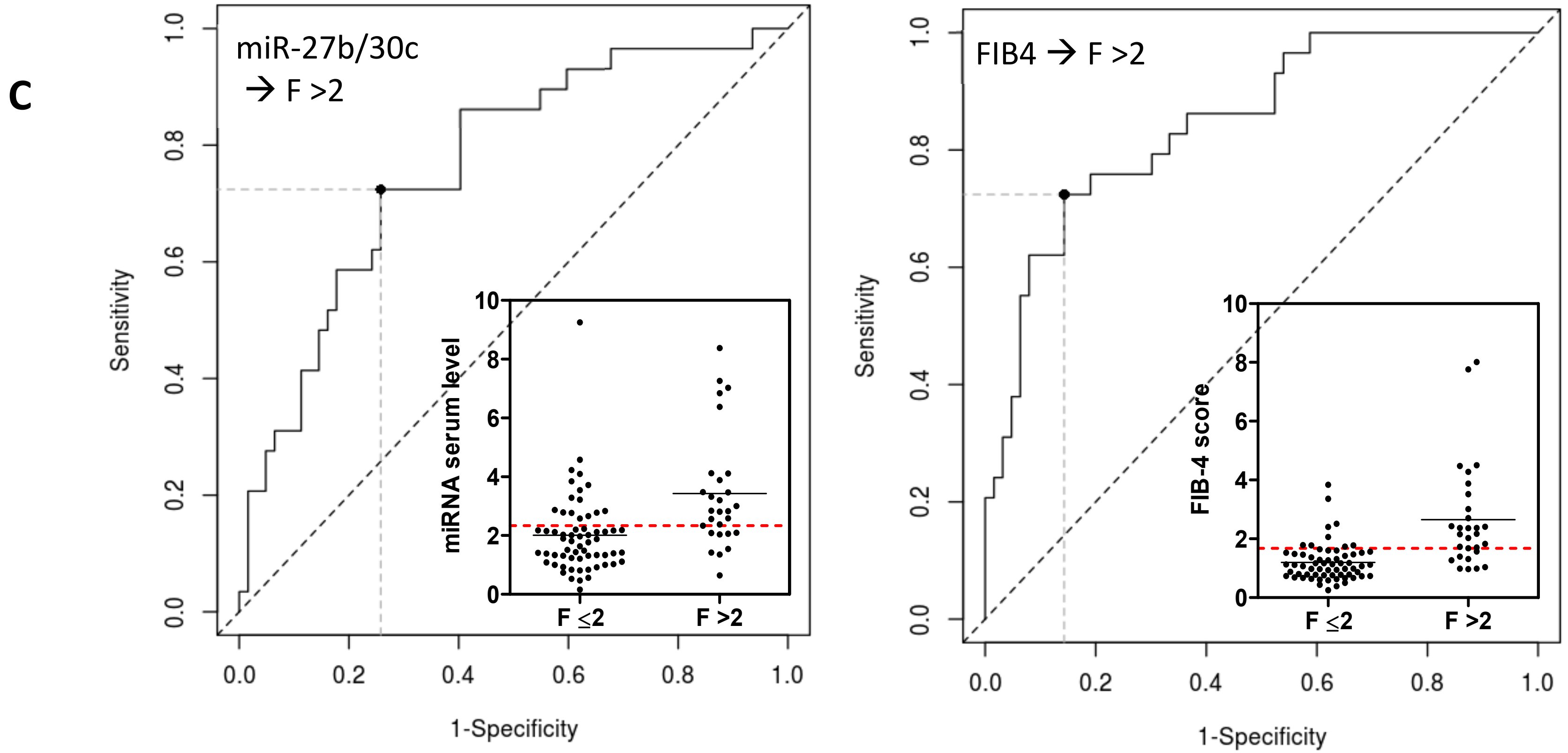

Supplementary Figure 1: **ROC curve analysis of predictors for severe NAFLD**. Selected miRNA ratios and conventional clinical markers and algorithms were tested as predictive biomarkers to discriminate patients by SAF Activity ≥2 **(A)**, NAS score ≥5 **(B)** or Fibrosis grade >2 **(C)**. Inserts depict the separation of the different groups according with the optimal cut-off by Youden index.

Supplementary Figure 2

A. PLS-DA model built for SAF A≥2 using serum-based clinical variables and performance evaluation by LOO-CV.

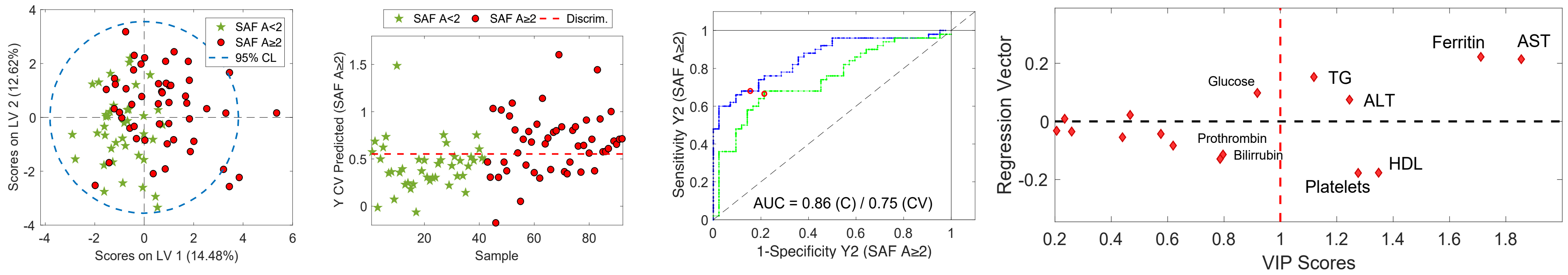

B. PLS-DA model built for SAF A≥2 using serum-miRNAs and performance evaluation by LOO-CV.

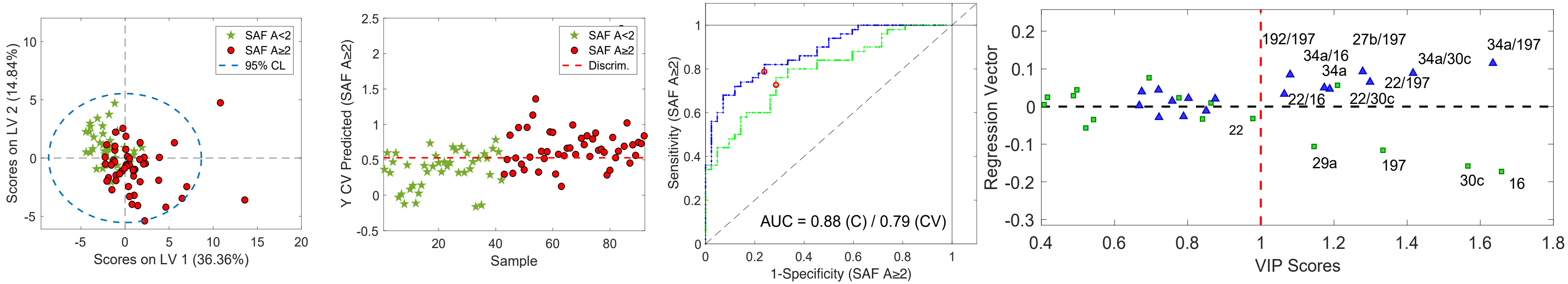

C. PLS-DA model built for SAF A≥2 using serum-based clinical and miRNA variables and performance evaluation by LOO-CV.

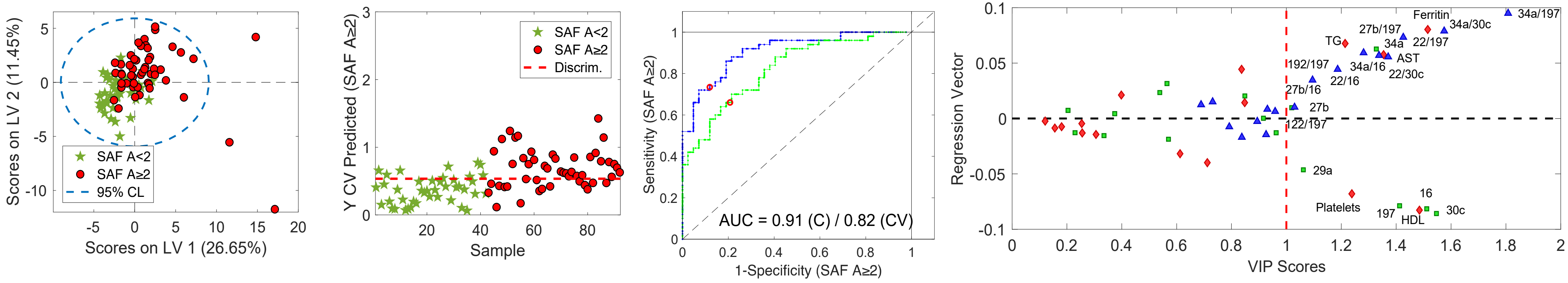

D. PLS-DA model built for NAS≥5 using serum-based clinical variables and performance evaluation by LOO-CV.

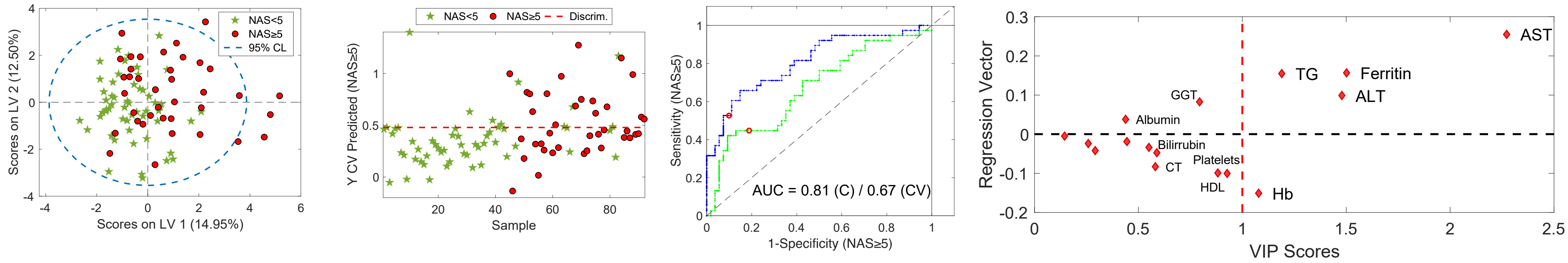

E. PLS-DA model built for NAS≥5 using serum-miRNAs and performance evaluation by LOO-CV.

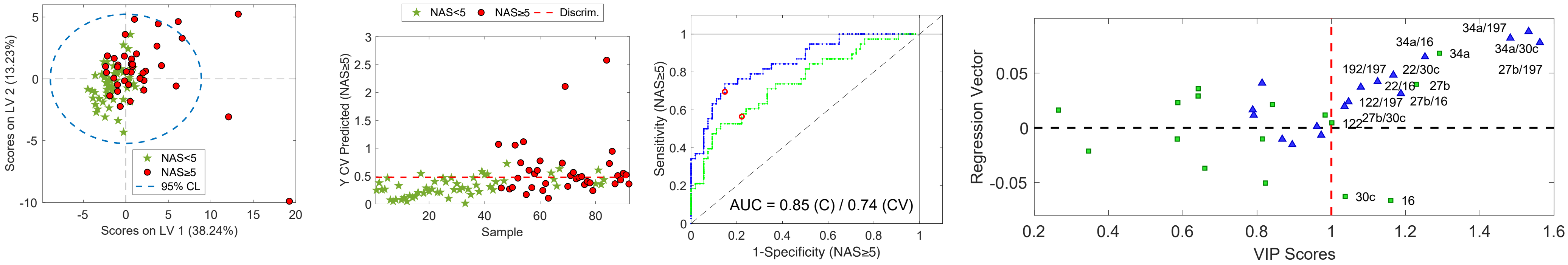

F. PLS-DA model built for NAS≥5 using serum-based clinical and miRNA variables and performance evaluation by LOO-CV.

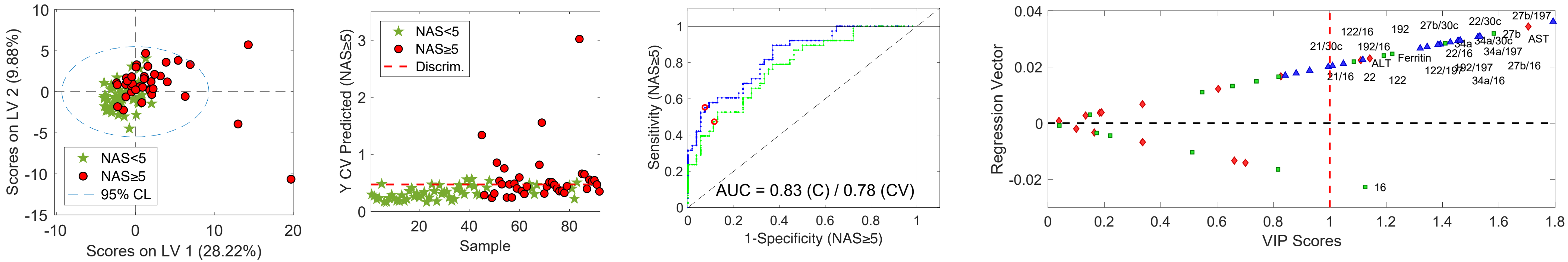

G. PLS-DA model built for F>2 using serum-based clinical variables and performance evaluation by LOO-CV.

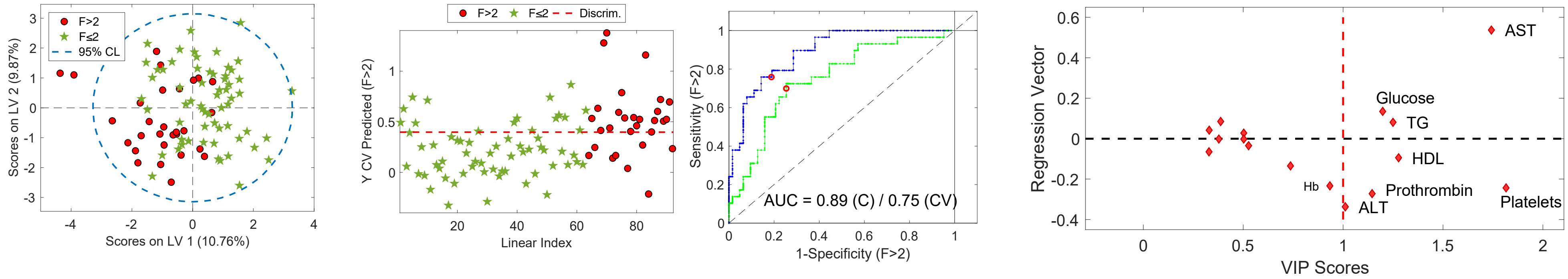

H. PLS-DA model built for F>2 using serum-miRNAs and performance evaluation by LOO-CV.

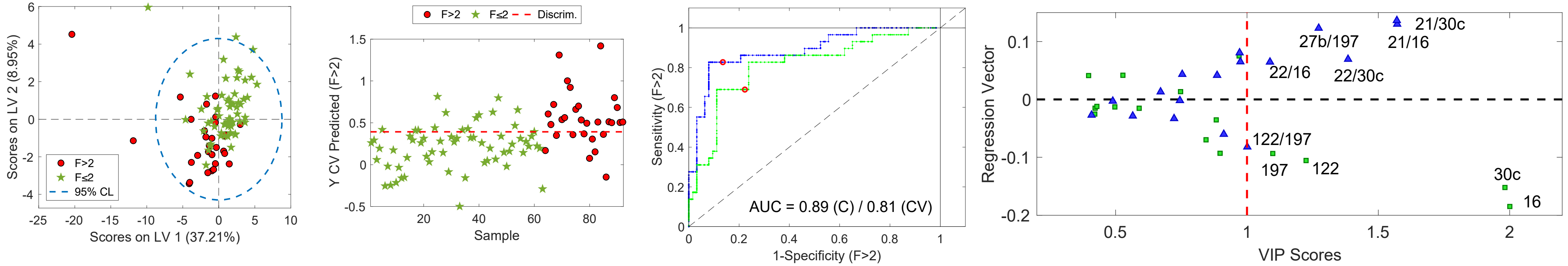

I. PLS-DA model built for F>2 using serum-based clinical and miRNA variables and performance evaluation by LOO-CV.

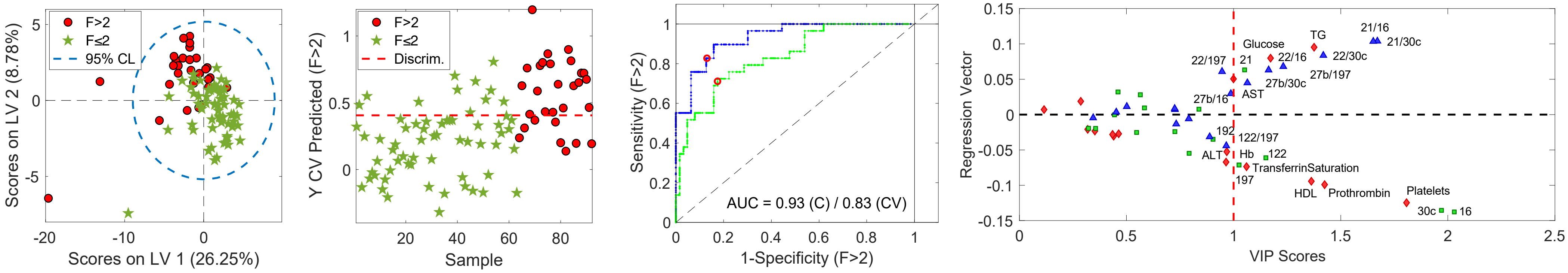

Supplementary Figure 2: **Multivariate PLS-DA models for the discrimination of SAF A ≥2 (A-C), NAS ≥5 (D-F) or F >2 (G-I) patients and model evaluation by leave-on-out cross validation (LOO-CV)**. For each panel, from left to right: 1) PLSDA scores plot showing the distribution of samples; 2) Predicted classes by LOO-CV. Samples with y CV predicted values above the discriminant threshold are classified as disease (i.e. SAF A ≥2, NAS ≥5 or F >2); 3) ROC curves estimated from calibration (blue line) and cross validation (green line) data; 4) Plot of the VIP scores and value in the regression of each variable included in the PLSDA model. The color indicated the type of variable (red: clinical; Green: miRNA; blue: miRNA ratio). A VIP score threshold equal to 1 is typically used to identify the most discriminant variables in a PLSDA model.
